# The crosstalk between EGF, IGF, and Insulin cell signaling pathways - computational and experimental analysis

**DOI:** 10.1186/1752-0509-3-88

**Published:** 2009-09-04

**Authors:** Rafal Zielinski, Pawel F Przytycki, Jie Zheng, David Zhang, Teresa M Przytycka, Jacek Capala

**Affiliations:** 1 National Cancer Institute National Institutes of Health Bethesda MD, USA; 2 Columbia College Columbia University New York, NY, USA; 3 National Center for Biotechnology Information, National Library of Medicine National Institutes of Health Bethesda, MD, USA; 4 Department of Electrical & Computer Engineering, University of Maryland, College Park, MD, USA

## Abstract

**Background:**

Cellular response to external stimuli requires propagation of corresponding signals through molecular signaling pathways. However, signaling pathways are not isolated information highways, but rather interact in a number of ways forming sophisticated signaling networks. Since defects in signaling pathways are associated with many serious diseases, understanding of the crosstalk between them is fundamental for designing molecularly targeted therapy. Unfortunately, we still lack technology that would allow high throughput detailed measurement of activity of individual signaling molecules and their interactions. This necessitates developing methods to prioritize selection of the molecules such that measuring their activity would be most informative for understanding the crosstalk. Furthermore, absence of the reaction coefficients necessary for detailed modeling of signal propagation raises the question whether simple parameter-free models could provide useful information about such pathways.

**Results:**

We study the combined signaling network of three major pro-survival signaling pathways: **E**pidermal **G**rowth **F**actor **R**eceptor (EGFR), **I**nsulin-like **G**rowth **F**actor-1 **R**eceptor (IGF-1R), and **I**nsulin **R**eceptor (IR). Our study involves static analysis and dynamic modeling of this network, as well as an experimental verification of the model by measuring the response of selected signaling molecules to differential stimulation of EGF, IGF and insulin receptors. We introduced two novel measures of the importance of a node in the context of such crosstalk. Based on these measures several molecules, namely Erk1/2, Akt1, Jnk, p70S6K, were selected for monitoring in the network simulation and for experimental studies. Our simulation method relies on the Boolean network model combined with stochastic propagation of the signal. Most (although not all) trends suggested by the simulations have been confirmed by experiments.

**Conclusion:**

The simple model implemented in this paper provides a valuable first step in modeling signaling networks. However, to obtain a fully predictive model, a more detailed knowledge regarding parameters of individual interactions might be necessary.

## Background

Signal transduction is the primary means by which cells respond to external stimuli such as nutrients, hormones, growth factors, and stress. Following the discovery of reversible phosphorylation [1] which provides the fundamental mechanisms of signal propagation, a large spectrum of methods that cells adopt to propagate a signal has been elucidated [2]. The discovery of basic principles of modular organization on the level of signaling domains [3] as well as on the level of whole signaling pathways (as exemplified by the MAP Kinase cascade which is present in multiple copies in eukaryotic organisms added to the understanding of the signaling process. These increasingly more accurate [2,4] descriptions of signaling mechanisms are accompanied with the reconstruction of ever larger signaling pathways and networks.

Defects in signaling pathways are associated with many serious diseases, in particular cancer [5]. Extensive molecular-level knowledge of signaling mechanisms raised expectation that such defects can be corrected with a therapeutic intervention using either receptor-specific antibodies or low-molecular weight compounds interfering with activation of the signaling molecules. However, individual signaling pathways do not act in isolation, but rather interact with each other, forming complex signaling networks that respond to diverse, often contradictory, stimuli. Such cross-talk can involve components that are common between pathways, as well as positive and negative feedback loops [2]. Furthermore, response to a signal depends on the activation threshold and signal duration [6] adding yet another level of complexity to the system. In consequence, a system level understanding of signaling networks is lagging behind the molecular-level knowledge of its constitutive components, which hinders a systematic approach to drug discovery.

The Epidermal Growth Factor (EGF) signaling pathway is one of the best understood receptor signaling pathways [7]. Members of the EGF receptor (EGFR) family have been shown to be overexpressed in several types of cancers and have been used as main targets for recently developed molecular therapies [8]. However, a significant fraction of cancers has been resistant to the current approach which is based on blocking individual growth factor receptors [9] suggesting that there may be crosstalk between EGF and other pro-survival pathways.

The interplay between the EGF pathway (and EGF plus Insulin) and its antagonist "pro-death" TNF signaling pathway has recently been a focus of a number of computational and experimental studies [10-17].

This work focuses on the signaling network formed by the interaction of three signaling pathways: Epidermal Growth Factor Receptor (EGFR), Insulin-like Growth Factor 1 Receptor (IGF-1R), and Insulin Receptor (IR). All three pathways are pro-survival and have been experimentally linked to cancer. Therefore, it is imperative for the development of successful targeted therapies aimed at these pathways that the crosstalk between them is well understood. In this work, we provide a combined computational and experimental analysis of this crosstalk.

The level of abstraction of a computational approach depends on the prior knowledge of the system and the type of experimental data [18]. We used a multi-level approach. First, we used a high level network model to discover basic properties of the network topology and to identify the nodes of the network that are most likely involved in the crosstalk. Next, we developed fuzzy Boolean network model to predict activities of nodes as functions of combinatorial stimulation of the EGF, IGF, and insulin receptors. That is we assume that each edge passes the signal with a certain probability. Consequently, rather than having binary active/inactive values, each node is active or not with a probability computed using a simulation protocol. In each step of the simulation, the probability of a node being active (i.e. activity level) is computed based on the activity values of its neighbors and the probabilities of transferring the signal along the corresponding edges. In general, different edges can have different probabilities (represented as edge weights); however, in this study we assign them equal values. Note that the method of signal propagation used in our approach is different from Petri net, which has been previously used to analyze signaling pathways [19]. Unlike Petri nets which require two types of nodes ("active" transitions and "passive" places), we use only one type of node. Each node computes the probability of activation using a Boolean function. In contrast, the transition nodes in Petri nets operate by passing units of information (the so called tokens).

The changes in activation of selected nodes following different levels of stimulation of these receptors were also measured experimentally. The experimental results were used to assess if the simple model was able to recover the general trends in the dependence between combinatorial stimulation and the level of activation and to further adjust the parameters of the network.

For this study, EGF, IGF, and insulin pathway information provided by STKE was combined into one signaling network. Our results indicate that, despite its simplicity, the model is able to predict several general patterns of the response and thus is expected to provide a good starting point for investigating signaling networks. To obtain a fully predictive model, a more accurate parameterization is necessary either by acquiring the precise knowledge of kinetic parameters of individual interactions or, given a sufficient number of experimental measurements, by training the model to fit the data.

## Results and discussion

### Network building and its static analysis

**Figure 1:**
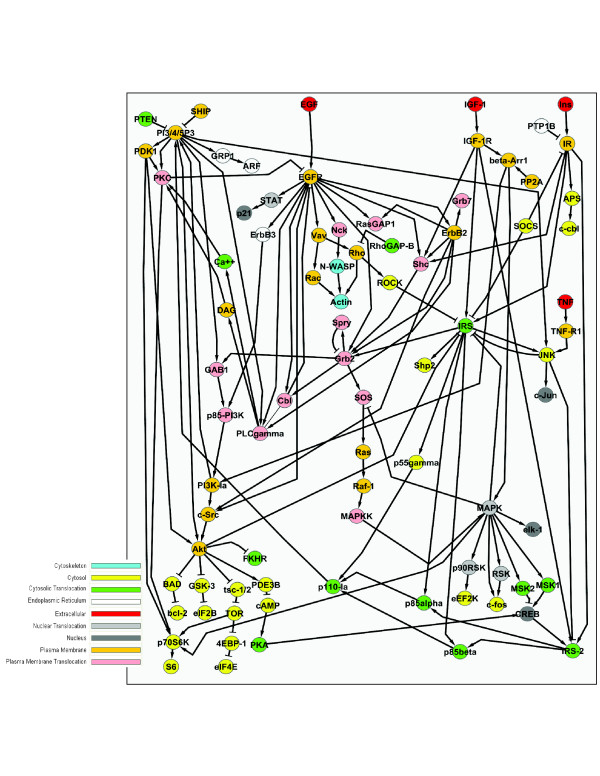
**Network used for static and dynamic analysis of combined EGFR, IGF-1R and IR signaling**. Figure drawn using Cytoscape [35]

We started by combining the information on EGF, IGF, and insulin pathways provided by STKE into one signaling network [20-22]. The resulting network comprises of 82 nodes representing receptor, cytosolic and nuclear proteins connected by stimulatory and inhibitory edges Figure 1. In this network, IGF-1R signaling was mediated only by β-arrestin. Therefore, to better reflect existing knowledge about this pathway in our network, we added three edges connecting IGF-1R to its well described adaptor proteins: IRS, IRS2 and Shc [23] (Figure 1).

In order to analyze the crosstalk between the three signaling pathways we experimentally monitored the activity of selected signaling molecules as a function of different activity levels of the three receptors. Monitoring of the activity of all molecules in the network would be most informative but, unfortunately, this is not feasible with current technology. This necessitated the development of a method for selection of the most informative set of molecules. An analysis of the topological properties of a biological network provides a good first view of the network as a whole and helps to identify components that play a central role in signal propagation. In the context of biological networks, two such measures attracted particular attention: the degree of the node and the betweenness centrality [24-26]. The degree of a node is the number of edges adjacent to the node, which is the number of interacting partners. The betweenness centrality is the sum over all pairs of nodes of the fraction of the shortest paths that go through a given node. Unfortunately, none of the above measures are directed towards identifying molecules most likely to be affected by the crosstalk between two or more sub-networks, thus they cannot be used to prioritize selection of the molecules such that measuring their activity would be most informative for understanding the crosstalk. Therefore, in this work we introduce two new measurements: network crosstalk and path crosstalk.

The *network crosstalk *of a node is the difference in the degree of the node in the network containing all considered pathways and the maximum degree of this node in any one individual pathway. A high *network crosstalk *value implies that a node is a branching node connecting two or more pathways.

**Figure 2:**
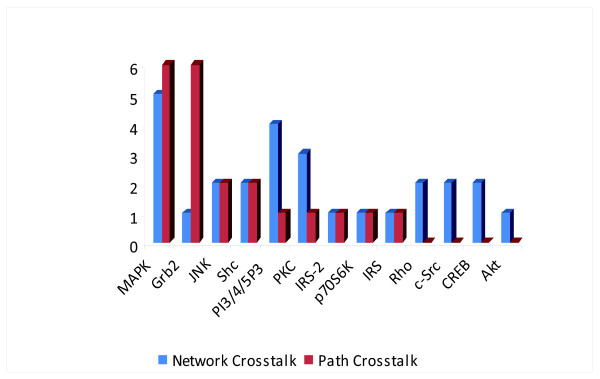
**The network crosstalk and path crosstalk values for all the signaling molecules in the network which have non zero network crosstalk value**.

To define the second measure, *path crosstalk*, we first introduce the signal-flow centrality. This measure considers the shortest paths going from all receptor molecules to all path-ending nuclear molecules. For each node, its signal-flow centrality equals the number of such paths going through it. We define the *path crosstalk of a node *as the difference between its signal-flow centrality for the entire network and its maximum signal-flow centrality in any one individual pathway. A high path crosstalk value suggests that a node is more important in the combined network than it was in the individual pathways. The network crosstalk and path crosstalk values for the signaling molecules which have non-zero network crosstalk are given in Figure 2.

The nodes with high network crosstalk correspond to the nodes where the signals from the diverse corresponding starting points are merged together while the nodes with high path crosstalk correspond to signaling molecules that are branching points for several pathways. We observed that the nodes indentified by this method as important for intra-pathway communication correspond to molecules that are assumed to be major crosstalk players in these well studied pathways. Most of these molecules are indicated as important in tumor genesis as well as other pathological states. This suggests that this approach provides a reasonable way of prioritizing experimental measurements for studies of a crosstalk between pathways and shall be useful for studies of less understood pathways.

### Computational analysis of networks response to differential receptors stimulation

To analyze the activity of the network in response to various levels of stimulation of the three receptors, we introduced a simple computational model. Similar to the standard representation of signaling pathways, the network is represented as a directed graph with two types of edges: activation edges and inhibition edges. However, the nodes as well as the edges have weights. This modification allows to replace the standard Boolean logic model [27] with fuzzy logic - a generalization of the standard Boolean logic that handles the concept of partial truth. Using such generalization is justified by the fact that the nodes in a signaling network typically represent assemblies of individual molecules each of which could be active or not. Consequently, each node is associated with a weight between 0 and 1 reflecting the level of activation of a given molecule (percentage of active molecules). In an iterative procedure (see Methods section) the activity of each node is updated based on the activities of its neighbors. In contrast to the node weights, the edge weights are not computed but included as a part of the input. The weight of an edge is a value between 0 and 1 representing the probability (efficiency) with which the signal is propagated along this edge. The necessity for modeling such signal loss was discovered by the comparison of the simulation results with edge weights set to one with the experimental results (data not shown). The trends observed for edge weights less than 1.0 had a much better agreement with the experimental results. Consequently, we used 0.8 as the default setting for all edge weights. In the next section we show the results for two different setting of weights. Obviously it is possible, and desirable if supported by a sufficient amount of experimental data, to assign different weights to different edges. However, to avoid any over-fitting in our simulation, we assigned non-default weights only to a small number of edges where such modification could be justified based on literature. We describe these modifications in the experimental validation section.

**Figure 3:**
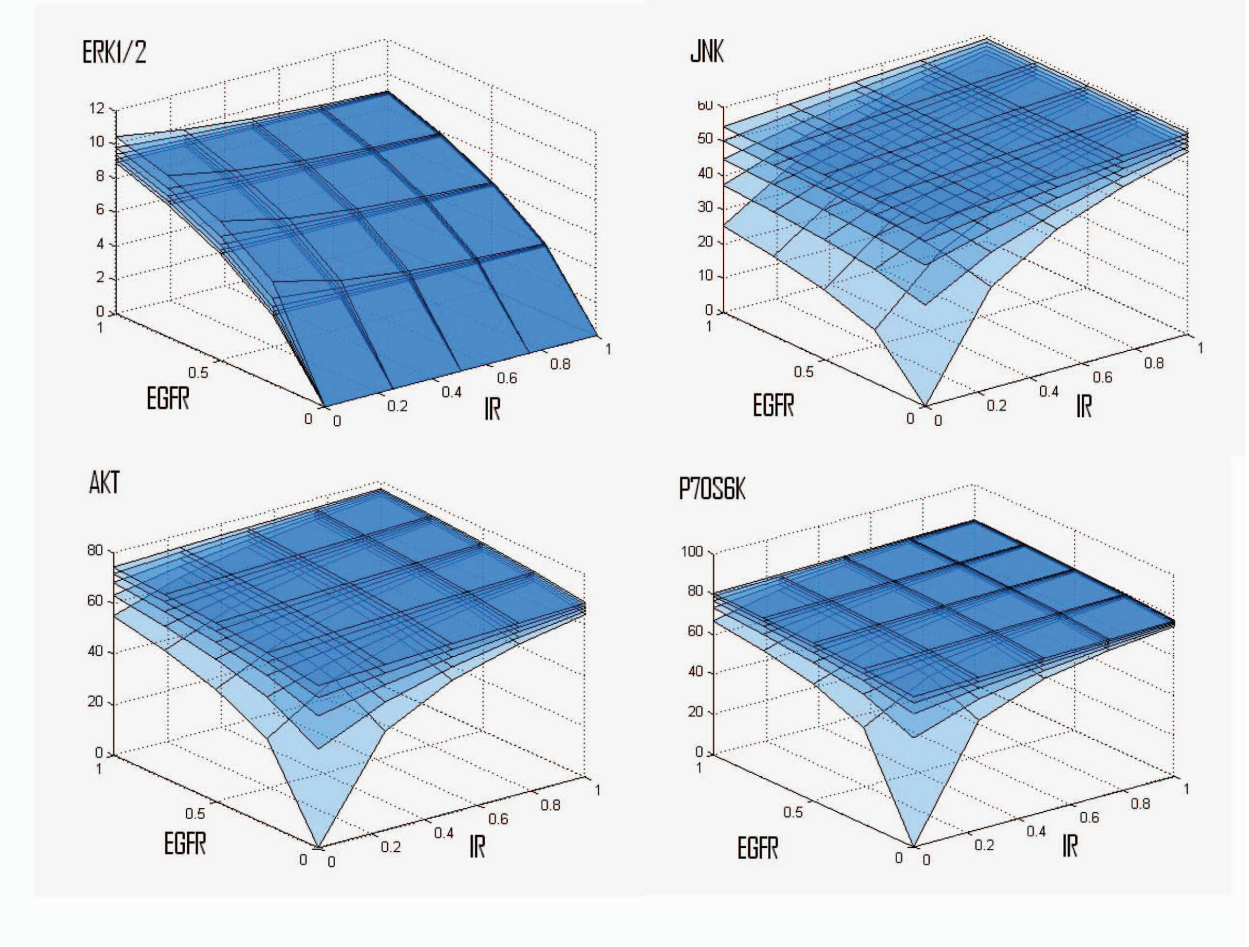
**The results of dynamic simulations for the four monitored molecules**. Each layer on the graph is a different level of activation of IGF-1R, with top layers corresponding to higher IGF-1R activities. Signal propagation for all edges was set at 0.8, except for the edges IGF-1R-Shc, IR-Shc, IRS-Shc and IGF-1R-β-arrestin whose weights were set as close to zero.

The default initial setting of all nodes is zero with the exception of the values for the receptors: EGF Receptor (EGFR), IGF Receptor (IGF-1R), and Insulin Receptor (IR). In the simulation we used five different levels of activation of these receptors: 0%, 25%, 50%, 75%, and 100%. Every possible combination of activation levels was simulated resulting in 125 different settings of the input parameters. The results of the simulation for the four molecules selected for experimental measurements: Erk1/2, Akt1, Jnk, and p70S6K, are given in Figure 3. As illustrated in Figure 2 all of them have "non zero" network crosstalk. Moreover, ERK1/2 and pJNK has also high path crosstalk value. All chosen molecules are downstream kinases, activated through phosphorylation, and influencing cell fate through modification of transcription and translation factors or direct phosphorylation of survival/apoptotic pathways components.

The results for the remaining molecules with non-zero network crosstalk are given in Additional file 1: Figure S1.

In computational simulation, all selected molecules showed dynamic dependency on the activation levels of at least two receptors. This was also true for all other molecules with non-zero network crosstalk (data not shown) as it would be expected based on the definition of network crosstalk. For Jnk, the dependence on EGFR activation is not very strong except near the point (0,0,0) while the activity of P70S6K is relatively stable for all three parameters (again, except near the point (0,0,0)). For Erk1/2 the strongest influence results from the variability in EGFR activation, while for Akt1 the variability in the activation of any of EGFR or IR has a similar effect which is also quite similar to the effect of IGF-1R.

As demonstrated in Figure 3, ERK1/2 activity was predicted not to depend on IR stimulation (Figure 3A), while EGFR activation was predicted to lead to activation of ERKs. The effect was even more pronounced when the cell was co-stimulated with IGF-1R. However, IGF-1R activation alone was predicted not to contribute to ERK1/2 activity as shown in Figure 3A.

Activation of the other MAPK family kinase JNK was predicted to depend strongly on all three receptors tested. According to simulation, the maximum activation level is observed after saturation of all receptors. EGFR was predicted to have the lowest impact. IR and IGF-1R applied alone at the same conditions activated JNK (Figure 3B).

Simulations of AKT kinase suggested that its activation depends on all tested receptors. Remarkably, efficient activation is achieved by stimulation and co-stimulation of IR and IGF-1R, while saturation of EGFR alone results in AKT activation at a lower extent. However, while activity of AKT increases rapidly with activation of either IGF-1R or EGFR and achieves levels close to the maximal, the saturation of both IGF-1R and EGFR seems necessary to maintain the highest AKT activation level. Under these conditions, IR seems to have the lowest impact (Figure 3C).

Our simulations also suggested a strong response of P70S6K to the activation of the three tested receptors. Even low activation of EGFR, IGF-1R and IR saturates p70S6K activity and further stimulation of receptors has a minimal effect of p70S6K activation (Figure 3D).

As discussed in the next section most (but not all) of these trends were confirmed experimentally.

### Experimental validation

Four proteins with non-zero network crosstalk, Erk1/2, Akt1, P70S6K and Jnk, were selected for experimental validation of our simple model and to obtain a set of quantitative data to guide necessary adjustments of the model. All molecules are indicated as important in tumorigenesis as well as other pathological states e.g. diabetes. All of them are well studied and tools for measuring their activation are readily available.

For the experimental study we used SKOV3 cell line. Receptor expression analysis of SKOV3 cells confirmed expression of EGFR, IGF-1R, and IR (data not shown). In order to compare experimental results with our computational results, we next measured the saturation levels of receptors in response to incubation with EGF, IGF and Ins (Additional file 1: Table S1). We also measured the cross-stimulation of IGF-1R and IR by their ligands, so that the corresponding correction could be included into simulation (Additional file 1: Table S1). This set of experiments allowed us to translate ligand dosage into activation level. Five sets of experiments were performed step-wise increasing the receptors activation by 25%. Using Mesoscale Discovery platform plates we measured the response to activation of individual receptors (Additional file 2: Table S1) and their co-stimulation in combination with each other (Additional file 2: Table S3). The results were reported as the percentage of phosphorylation of each examined molecule.

**Figure 4:**
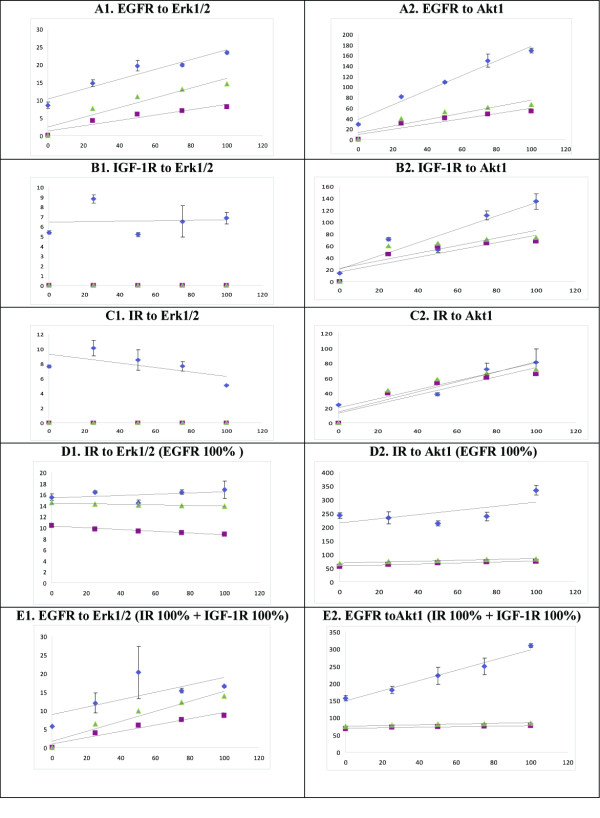
**Comparison between experimental and simulated data**. In the figures in the left column, y axes are activities of Erk1/2; In the figures in the right column, y axes are activities of Akt1. In each figure, blue diamonds represent real data; green triangles represent the results of simulation, where we set weights of both activation and blockage edges to 0.8; red squares represent the data of simulation, where we set weights of activation edges to 0.8 and blockage edges to 0.5. The caption above each figure indicates the x and y variables. Figures of D and E report the results for the 100% stimulation of remaining receptors. In simulation, we also included a correction for the cross-stimulation between IR and IGF-1R, according to Table S1.

We observed that computer simulations were able to recapitulate most of trends observed in the experimental studies. In particular, increasing Akt1 activity in response to receptor stimulation was confirmed for all tested combinations. The highest Akt1 activation level was observed when cells were co-stimulated with all three ligands simultaneously (Additional file 2: S3). As predicted, increased EGFR stimulation translated to increased Erk1/2 activity. However co-stimulation of the receptors with insulin and/or IGF did not lead to increase in Erk1/2 activity. Specifically, we didn't observe a dependency of the activity of Erk1/2 on activation level of IR and IGF-1R. This was not in the agreement with our initial model derived directly from the STKE data. However, this counterintuitive observation could be explained in light of the findings reported by Yuhong Lu *et al*. 2003 [28] The authors showed that in breast cancer cells with high levels of HER2 expression, signaling from IGF to Erk1/2 was attenuated, and this inhibition was reversed after the number of HER2 had been lowered by treatment with shRNA. They also showed that the overexpression of HER2 increased the baseline level of Shc phosphorylation and the association of Shc with Grb2, but reduced the IGF induced Shc phosphorylation, the IGF induced Shc association with Grb2, and consequently the Erk1/2 activation. They formulated the hypothesis that induction of Shc phosphorylation and its association with Grb2 by HER2 overexpression might result in a reduction of free Shc and Grb2 available upon IGF-1R activation in these cells. Since SKOV3 cells express high level of HER2, we tried to apply the same explanation to address observed discrepancies. To reflect this situation in our model, we reduced the weights of the IR-Shc, Irs-Grb2, and IGF-1R - Shc edges. This modification resulted in a significant decrease in Erk1/2 sensitivity to Ins stimulation and a better fit to the experimental data (Figure 3, Additional file 2: Table S3). However, this modification did not change the Erk1/2 response to IGF (data not shown). To elucidate the reasons for the above inconsistency, we repeated the computational signal flow analysis and we found out that the only node able to mediate the signal flow from IGF-1R to Erk1/2 is β-arrestin. This pathway was reported by Lefkovitz's group in 2003 and has been included in Science Signaling [29]. The authors showed that IGF-bound receptors can signal through β-arrestin in a kinase independent manner resulting in Erk1/2 activation in melanoma cells [30] and Akt1 in mouse embryo fibroblast (MEF) [31]. It appears, however, that in SKOV3 cells, at least in our experimental setting, β-arrestin does not play the crucial role in signal transduction from EGFR to Erk1/2. Therefore, in order to better fit the experimental data, we decreased the probability of the IGF-1R-β-arrestin edge. This resulted in a reduced response of Erk1/2 to IGF stimulation (Figure 4.B1).

Figure 4 shows all experimental measurements alongside with simulation results after the modifications for Akt1 and Erk1/2 as described above. The computational results are shown for two parameter setting of all remaining edges: (i) all edges not discussed above are set to 0.8, (ii) all activation edges set as in (i), while all inhibition edges set to 0.5. It is clear that, while the slopes of the regression lines are different in both setting of the parameters, the general trend remains the same. Table Additional file 1: Table S1 and Additional file 2: Table S3 provides full list the results of experimental and computational analysis for the first parameter setting.

However, a few discrepancies remained unresolved. For example, while in agreement with simulation results, experimental data showed weak increase in p70S6K activity after stimulation with EGF, no such simulation-predicted increase was obtained experimentally for Ins and IGF. It appears that, in SKOV3 cells, the p70S6K activation is affected only by EGF. Similar inconsistency is observed for Jnk. Interestingly, despite the fact that it is well established that Jnk kinase mainly responds to stress stimuli and inflammatory cytokines, there are reports pointing out induction of Jnk activity after stimulation with growth factors [32,33]. Such trends are also predicted by our computational model. However, in our experiments, stimulation with different combination of growth factors did not cause increase in T183 and Y185 phosphorylation of Jnk (Additional file 2: Table S2). Again, it is possible that significant overexpression of HER2 causes perturbation in the Jnk activation pathway in a way that is not captured by our model. Additional studies are required in order to resolve these discrepancies.

## Conclusion

It is now understood that traditionally defined singling pathways are not functioning in isolation but rather form whole signaling networks involving crosstalk between individual pathways. Can such crosstalk be studied in the absence of high throughput technology that would allow measuring reaction rates, association constants, and other parameters needed to faithfully model a signaling network? We addressed this question on two levels. First, given that we deal with low throughput, labor intensive technologies, we needed to have a measure that would prioritize experimental measurements. To achieve this goal, we developed two measures of involvement of a node in a crosstalk. These measures are based on static, topological properties of the network and are intuitive extensions of centrality measures used to asses essentiality of proteins in protein-protein interaction networks. Subsequently, we used so defined crosstalk measures to facilitate the selection of relevant molecules for computational and experimental evaluation.

The second question that we addressed in this work was whether a simple model that lacks specific data such as abovementioned reaction rates, association constants, etc. can predict activity of a network in response to various levels of stimuli. Consequently, we developed a simple model and tested it on the network constructed by combining EGF, IGF and insulin signaling pathways. Despite its simplicity, the model was in agreement with most experimentally observed trends. It proved to be a valuable tool in the initial investigation of the signaling network in SKOV3 cells. Specifically, the examination of the discrepancy between the expected and obtained results pointed to possible differences between signaling in the studied SKOV3 cells and the available canonical signaling pathways. We confirmed some of these discrepancies through a literature search and subsequently corrected the model.

Intuitively, one can see our model as a simple extension of the standard Boolean-network type of model by adding a stochastic component to it. This view is intuitive but it has not been, until now, confronted with reality. Interestingly, we found that a necessary element of the model is the assumption that the signal is not propagated with 100% efficiency. Without this assumption, the model was unable to correctly predict most of the trends.

Our study indicate, that while crude models, such as the one exercised in this paper, cannot substitute for more precise simulation, they can still provide valuable information on the response of signaling network to stimuli. As more experimental data is being gathered, the parameters of the models will be adjusted to fit the data. It is also possible (and quite likely) that our network is incomplete. However given more data points one can start to employ Bayesian methods to fill the missing edges and improve this aspect of the model.

## Methods

### Experimental procedures

Human ovary carcinoma, SKOV3, cells were cultured in McCoy medium supplemented with FBS to the final concentration of 10% at 37°C in 5% CO_2_. For every experiment, five 10 cm Petrie Dishes were plated with 2 × 10^6 ^cells each to obtain 90% of confluence fifteen hours later. Cells were then starved for 24 hours in McCoy with 0.5% FBS and subsequently stimulated with different combination of EGF, IGF and Ins for 15 min. After the 15-min stimulation the cells were immediately rinsed two times with ice-cold PBS and lysed in complete Mesoscale Discovery (MSD) lyses buffer containing complete mix of proteases and phosphatases inhibitors. After 20 min of incubation cells were sonicated in four 10-second intervals, followed by a cooling step. Finally, the cell lysates were centrifuged at 15000 G for 20 min, aliquoted in small volumes, flash frozen, and stored at -80°C for further analyses. Protein phosphorylation levels were measured using signaling MAPK and Akt1 panel phospho/total assay. EGFR saturation level was determined using EGFR activated/total duplex kit. Saturation of IR and IGF-1R was measured using Insulin signaling phospho/total assay. All assays were purchased from Mesoscale Discovery platform; Gaithersburg, MD and the measurements were carried out according to manufacturer protocols. Each measurement was performed in triplicates. Protein phosphorylation was calculated as the ratio of signal of "phosphorylated" form to it's "total" counterpart" multiplied by factor 100.

### Computational procedures

The network simulation was performed by iteratively traversing the nodes of the network using a Breadth First Search (BFS) approach [34]. Each BFS traversal started from the receptors and propagated the signal towards the terminal nodes. The activity of each node is determined from the activities of its neighbors in the previous step as described below. The initial values of the activities of all nodes were set to zero with the exception of the receptor activities where every possible combination of each level of activation (0%, 25%, 50%, 75%, and 100%) was used and kept constant during the simulation. Since in this computation we are interested in estimating the steady state activation levels of these molecules in response to various activation levels, the fact that we used BFS order was not critical (although it is useful for applications of our software to model response to a short impulse rather than prolonged stimulation). Let *u *be the node to be updated and let *A*_*t*_(*i*) denote the activity of node *i *as computed in the *t*-th iteration and let *w*(*i*, *j*) be the weight of edge (*i*, *j*). Let *S *be the set of neighbors of *u *that are activators and *B *be the set of repressors. Informally we compute the probability of node *u *being activated by at least one activator ("or" function on activators) and not being repressed by any of the repressors ("and" function on repressors). By De Morgan's laws, *A*_*t*_(*u*) is computed as follows:



For comparison with experiments we used averaged results of 100 iterations.

The simulation program is currently available on request and a version with enhanced functionality, graphical displays, and appropriate graphical user interface will be shortly publically available as a Cytoscape plugin [35].

## Authors' contributions

RZ performed the wet lab experiments, participated in discussions and writing the manuscript; PFP performed static and dynamic analysis, wrote programs for the static and dynamic analysis, drafted the initial manuscript, and participated in wet lab experiments; JZ performed additional static and dynamic analysis, participated in discussions and writing the manuscript; DZ wrote Cytoscape plugin and contributed Figure 1; TMP and JC designed and supervised the project, participated in discussions and writing the manuscript. All authors have read and approved the final manuscript.

## Supplementary Material

Additional file 1 **Phosphorylation level of receptors following stimulations with respective ligands in SKOV3 cells**. Estimation of receptor phsosporylation as the function of ligand concentration. Estimation of Cross-stimulation of IGF-1R and IR Click here for file

Additional file 2 **Phosphorylation of Erk1/2, Jnk, Akt1, P70S6K following stimulation and co-stimulation of EGFR, IGF-1R, and IR**. Tables of phosphorylation levels of four "output" protein in response to 25%- wise increase of receptor saturation along with simulation- predicted values. Click here for file
