# Supplementary material for: The crosstalk between EGF, IGF, and Insulin cell signaling pathways - computational and experimental analysis
Source: BMC Syst Biol. 2009 Sep 4;3:88. doi: 10.1186/1752-0509-3-88 (PMC2751744; doi:10.1186/1752-0509-3-88)
Supplement: Additional file 1 — Phosphorylation level of receptors following stimulations with respective ligands in SKOV3 cells. Estimation of receptor phsosporylation as the function of ligand concentration. Estimation of Cross-stimulation of IGF-1R and IR [file 1752-0509-3-88-S1.doc]

**Additional file 1**

**File format**: DOC

**Title:** Phosphorylation level of receptors following stimulations with respective ligands in SKOV3 cells.

**Description:** Obtained data were used to set the input concentration of the ligands during experimental verification of the model. Cross-stimulation of IGF-1R and IR is taken into consideration.

**Figure S1**

Figure S1. Receptor saturations after stimulations with respective ligands. Ligand concentration corresponding to 50% of receptor saturation are given in boxes.

.

**Table S1**

Table S1. Ligands concentration and corresponding receptor saturation level in SKOV3 cells used in experimental verification of the model.

| **IGF-1R sat %** | **Concentration of IGF, ng/ml** | **IR sat %** |
| --- | --- | --- |
| 0% | 0.00 | 0.0 |
| 25% | 6.95 | 13.4 |
| 50% | 20.84 | 31.7 |
| 75% | 62.52 | 58.1 |
| 100% | 200.00 | 81.6 |
| **IR sat, %** | **Concentration of Ins, μg/ml** | **IGF-1R sat. %** |
| 0% | 0.00 | 0.0 |
| 25% | 0.05 | 1.4 |
| 50% | 0.15 | 4.2 |
| 75% | 0.45 | 11.6 |
| 100% | 1.50 | 30.4 |
| **EGFR sat. %** | **Concentration of EGF, ng/ml** |  |
| 0% | 0.00 | **-** |
| 25% | 4.12 | **-** |
| 50% | 12.36 | **-** |
| 75% | 37.08 | **-** |
| 100% | 120.00 | **-** |
