# Supplementary material for: The crosstalk between EGF, IGF, and Insulin cell signaling pathways - computational and experimental analysis
Source: BMC Syst Biol. 2009 Sep 4;3:88. doi: 10.1186/1752-0509-3-88 (PMC2751744; doi:10.1186/1752-0509-3-88)
Supplement: Additional file 2 — Phosphorylation of Erk1/2, Jnk, Akt1, P70S6K following stimulation and co-stimulation of EGFR, IGF-1R, and IR. Tables of phosphorylation levels of four "output" protein in response to 25%- wise increase of receptor saturation along with simulation- predicted values. [file 1752-0509-3-88-S2.doc]

**Additional file 2.** Phosphorylation of Erk1/2, Jnk, Akt1, and P70S6K following stimulation and co-stimulation of EGFR, IGF-1R, and IR.

Table S2 Activation of Erk1/2, Jnk, Akt1 and p70S6K in response to EGFR (A) IGF1-R (B) and (IR) stimulation. Exp. columns correspond to the experimental results and Sim. columns contain simulation results with parameter setting described in main text.

| Rec. Sat | **Erk1/2** | | **Jnk** | | **Akt1** | | **P70S6K** | |
| --- | --- | --- | --- | --- | --- | --- | --- | --- |
| **A EGFR** | | | | | | | | |
|  | Exp. | Sim | Exp. | Sim. | Exp. | Sim. | Exp. | Sim. |
| 0 | **8.53**±0.96 | **0.00** | **1.61**±0.14 | 0.00 | **28.47**±2.08 | 0.00 | **6.59**±0.48 | 0.00 |
| 25 | **14.79**±0.85 | **6.02** | **1.51**±0.14 | 15.39 | **81.2**7±1.38 | 33.77 | **8.7**3±0.79 | 48.04 |
| 50 | **19.61**±1.47 | **8.18** | **1.68**±0.09 | 20.22 | **108.35**±1.8 | 44.11 | **9.34**±0.22 | 58.20 |
| 75 | **19.89**±0.38 | **9.49** | **1.80**±0.016 | 23.08 | **149.48**±12.3 | 50.27 | **11.26**±2.41 | 63.36 |
| 100 | **23.31**±0.43 | **10.41** | **1.98**±0.08 | 25.09 | **168.66**±4.53 | 54.63 | **9.14**±1.29 | 66.61 |
| **B IGF-1R** | | | | | | | | |
| 0 | **5.39**±0.18 | **0.00** | **1.26**±0.05 | 0.00 | **14.36**±0.66 | 0.00 | **5.12**±0.04 | 0.00 |
| 25 | **8.80**±0.41 | **0.00** | **1.20**±0.11 | 35.59 | **70.86**±2.92 | 45.88 | **6.30**±0.39 | 61.02 |
| 50 | **5.17**±0.20 | **0.00** | **1.06**±0.10 | 47.37 | **53.64**±4.75 | 58.31 | **6.21**±0.57 | 71.06 |
| 75 | **6.49**±1.60 | **0.00** | **1.39**±0.22 | 54.08 | **111.00**±7.26 | 64.61 | **6.55**±0.15 | 75.24 |
| 100 | **6.84**±0.59 | **0.00** | **1.16**±0.06 | 57.60 | **134.45**±13.06 | 67.64 | **5.97**±0.20 | 77.06 |
| **C IR** | | | | | | | | |
| 0 | **7.63**±0.19 | **0.00** | **1.48**±0.06 | 0.00 | **24.33**±0.85 | 0.00 | **7.02**±0.39 | 0.00 |
| 25 | **10.11**±1.04 | **0.00** | **1.72**±0.12 | 30.23 | **94.31**±4.86 | 39.72 | **5.89**±0.23 | 55.10 |
| 50 | **8.49**±1.39 | **0.00** | **1.67**±0.15 | 42.06 | **38.37**±2.08 | 52.92 | **6.20**±0.07 | 67.01 |
| 75 | **7.65**±0.63 | **0.00** | **1.40**±0.09 | 49.54 | **71.82**±8.45 | 60.42 | **5.73**±0.13 | 72.53 |
| 100 | **5.08**±0.100 | **0.00** | **0.98**±0.09 | 54.99 | **81.31**±17.79 | 65.41 | **5.92**±0.16 | 75.74 |

Table S3. Activation of Erk1/2, Jnk, Akt1 and p70S6K in response to co-stimulation of EGFR and IR (A), EGFR, IR and IGF-1R (B). Exp. columns correspond to the experimental results and Sim. columns contain simulation results with parameter setting described in main text.

| IR | Erk1/2 | | Jnk | | Akt1 | | P706SK | |
| --- | --- | --- | --- | --- | --- | --- | --- | --- |
| **A** | | | | | | | | |
| **IR %** | **EGFR 100% saturation** | | | | | | | |
|  | Exp. | Sim. | Exp. | Sim. | Exp. | Sim. | Exp. | Sim. |
| 0 | **15.48**±0.59 | 10.41 | **1.55**±0.27 | 25.09 | **242.18**±10.17 | 54.63 | **9.35**±0.74 | 66.61 |
| 25 | **16.43**±0.21 | 9.74 | **1.69**±0.08 | 37.39 | **232.65**±22.08 | 63.06 | **9.26**±0.63 | 73.40 |
| 50 | **14.48**±0.52 | 9.32 | **1.48**±0.23 | 45.17 | **213.18**±8.89 | 68.26 | **9.53**±0.57 | 76.97 |
| 75 | **16.43**±0.43 | 9.03 | **1.59**±0.07 | 50.93 | **238.75**±15.74 | 71.98 | **9.71**±0.23 | 79.27 |
| 100 | **16.86**±1.59 | 8.80 | **1.54**±0.17 | 55.48 | **333.53**±17.47 | 74.79 | **9.81**±1.35 | 80.87 |
| **B** | | | | | | | | |
| **EGFR** | **IR and IGF-1R 100%** | | | | | | | |
| 0 | **5.68**±0.06 | 0.00 | **1.26**±0.05 | 58.44 | 156.89±8.31 | 68.33 | **6.37**±**0.11** | 77.45 |
| 25 | **12.00**±2.66 | 3.83 | **1.20**±0.11 | 58.48 | 180.78±10.69 | 71.39 | **9.20**±**0.32** | 79.28 |
| 50 | **20.22**±7.04 | 6.04 | **1.06**±0.10 | 58.50 | 221.57±25.41 | 73.54 | **9.37**±**0.99** | 80.42 |
| 75 | **15.33**±0.66 | 7.55 | **1.39**±0.22 | 58.51 | 249.01±24.07 | 75.21 | **9.26**±**0.41** | 81.23 |
| 100 | **16.47**±0.38 | 8.67 | **1.16**±0.06 | 58.51 | 309.37±6.01 | 76.59 | **8.39**±**0.82** | 81.84 |
